# Short-Term Changes in Respiratory Biomarkers after Swimming in a Chlorinated Pool

**DOI:** 10.1289/ehp.1001961

**Published:** 2010-09-12

**Authors:** Laia Font-Ribera, Manolis Kogevinas, Jan-Paul Zock, Federico P. Gómez, Esther Barreiro, Mark J. Nieuwenhuijsen, Pilar Fernandez, Carolina Lourencetti, Maitane Pérez-Olabarría, Mariona Bustamante, Ricard Marcos, Joan O. Grimalt, Cristina M. Villanueva

**Affiliations:** 1 Centre for Research in Environmental Epidemiology, Barcelona, Spain; 2 Municipal Institute of Medical Research, IMIM-Hospital del Mar, Barcelona, Spain; 3 CEXS (Ciències Experimentals i de la Salut), Universitat Pompeu Fabra, Barcelona, Spain; 4 CIBER Epidemiología y Salud Pública, Barcelona, Spain; 5 National School of Public Health, Athens, Greece; 6 Hospital Clínic-IDIBAPS (Institut d’Investigacions Biomèdiques August Pi i Sunyer), Barcelona, Spain; 7 CIBER de Enfermedades Respiratorias, Bunyola, Mallorca, Spain; 8 Pulmonology Department-URMAR (Unitat de Recerca en Múscul i Aparell Respiratori), IMIM-Hospital del Mar, Barcelona, Spain; 9 Institute of Environmental Assessment and Water Research, CSIC (Consejo Superior de Investigaciones Científicas), Barcelona, Spain; 10 Department of Genetics and Microbiology, Universitat Autònoma de Barcelona, Barcelona, Spain

**Keywords:** biomarkers, Clara cell secretory protein, disinfection by-products, exhaled breath condensate, fractional exhaled nitric oxide, respiratory health, swimming, swimming pools, trihalomethanes

## Abstract

**Background:**

Swimming in chlorinated pools involves exposure to disinfection by-products (DBPs) and has been associated with impaired respiratory health.

**Objectives:**

We evaluated short-term changes in several respiratory biomarkers to explore mechanisms of potential lung damage related to swimming pool exposure.

**Methods:**

We measured lung function and biomarkers of airway inflammation [fractional exhaled nitric oxide (FeNO), eight cytokines, and vascular endothelial growth factor (VEGF) in exhaled breath condensate], oxidative stress (8-isoprostane in exhaled breath condensate), and lung permeability [surfactant protein D (SP-D) and the Clara cell secretory protein (CC16) in serum] in 48 healthy nonsmoking adults before and after they swam for 40 min in a chlorinated indoor swimming pool. We measured trihalomethanes in exhaled breath as a marker of individual exposure to DBPs. Energy expenditure during swimming, atopy, and *CC16* genotype (rs3741240) were also determined.

**Results:**

Median serum CC16 levels increased from 6.01 to 6.21 μg/L (average increase, 3.3%; paired Wilcoxon test *p* = 0.03), regardless of atopic status and *CC16* genotype. This increase was explained both by energy expenditure and different markers of DBP exposure in multivariate models. FeNO was unchanged overall but tended to decrease among atopics. We found no significant changes in lung function, SP-D, 8-isoprostane, eight cytokines, or VEGF.

**Conclusions:**

We detected a slight increase in serum CC16, a marker of lung epithelium permeability, in healthy adults after they swam in an indoor chlorinated pool. Exercise and DBP exposure explained this association, without involving inflammatory mechanisms. Further research is needed to confirm the results, establish the clinical relevance of short-term serum CC16 changes, and evaluate the long-term health impacts.

Swimming is practiced extensively in Western countries (Vaz et al. 1999). Despite the benefits of physical activity, health concerns are growing because swimming in pools involves exposure to disinfectants and disinfection by-products (DBPs), such as trihalomethanes (THMs), one of the classes of DBPs at highest concentration in swimming pools, and trichloramine, a known irritant (World Health Organization 2006). A range of acute symptoms has been described among bathers after accidental exposure to high levels of chlorine in swimming pools, including mucosal and ocular irritation, cough, rash, dyspnea, and lung function decline (Bonetto et al. 2006; Grasemann et al. 2007). Subjects exposed chronically to the swimming pool environment, such as pool workers, showed irritant eye, nasal, and throat symptoms (Jacobs et al. 2007; Massin et al. 1998). Cases of occupational asthma and trichloramine sensitization have been described in pool lifeguards (Thickett et al. 2002). Although an increased asthma risk among children attending pools has been suggested but not confirmed (Font-Ribera et al. 2009; Goodman and Hays 2008), respiratory symptoms and asthma are consistently more prevalent among competitive swimmers compared with other athletes (Goodman and Hays 2008). However, one of the unsolved questions is what are the biological mechanisms behind these health effects (Bonetto et al. 2006; Grasemann et al. 2007).

The development of methods to evaluate respiratory and systemic biomarkers in blood, exhaled breath condensate (EBC), and exhaled breath has allowed the assessment of pathobiological mechanisms underlying respiratory disorders (Bonetto et al. 2006) and the detection of early subclinical respiratory effects after acute or chronic environmental exposures, including swimming pool attendance. Lung surfactant proteins, such as Clara cell secretory protein (CC16) or surfactant protein D (SP-D), are secreted in the lung epithelium and move passively across the epithelial barrier into the serum down a strong gradient (Broeckaert et al. 2000). A change in the concentration of lung surfactant proteins in serum has been proposed as a marker to detect early permeability changes in the lung epithelium (Broeckaert et al. 2000). Fractional concentrations of orally exhaled nitric oxide (FeNO) is a marker of eosinophilic airway inflammation (Choi et al. 2006) and has been shown to increase after short-term exposure to mold (Stark et al. 2005). Soluble molecules can be detected in EBC, including proinflammatory cytokines, growth factors, and oxidative stress biomarkers, and have been used to monitor different aspects of diseases such as asthma or chronic obstructive pulmonary disease, as well as the effects of environmental stressors or physical exercise (Bonsignore et al. 2003; Carbonnelle et al. 2002, 2008; Massin et al. 1998; Nanson et al. 2001).

Proposed mechanisms of respiratory damage related to swimming pool exposure include airway inflammation (Bonetto et al. 2006; Grasemann et al. 2007; Moreira et al. 2008; Pedersen et al. 2009), oxidative stress (Varraso et al. 2002), and hyperpermeability of the lung epithelium (Bonetto et al. 2006; Carbonnelle et al. 2002, 2008). Increased permeability of the lung epithelium has been evaluated extensively, and some authors suggest that it may result in increased airway inflammation and higher risk of sensitization and allergic diseases (Bernard 2007). The role of previous atopic status is unclear because some studies have found higher asthma risk for swimming pool attendance among atopics (Bernard et al. 2006, 2007, 2008), but another has not (Font-Ribera et al. 2009). Few studies have measured lung function and respiratory biomarkers after swimming in chlorinated pools (Carbonnelle et al. 2002, 2008; Moreira et al. 2008; Pedersen et al. 2009). Lung function and FeNO were consistently unaltered in the studies, but contradictory results were obtained for lung epithelium permeability, estimated with serum levels of surfactant proteins. Consequently, the evidence remains inconclusive and inconsistent for some biomarkers, too.

The aim of this study was to explore short-term respiratory changes in healthy adults after swimming in an indoor chlorinated swimming pool by measuring lung function and a wide range of biomarkers that may reflect different mechanisms of effect, specifically, airway inflammation (FeNO, eight cytokines, and a growth factor in EBC), oxidative stress (8-isoprostane in EBC), and epithelial lung permeability (SP-D and CC16 in serum), taking into account both exposure to DBPs and physical exercise. Associations between swimming and these outcomes may provide clues regarding potential mechanisms through which swimming-related exposures might affect respiratory health.

## Materials and Methods

### Design

The study has a crossover design involving 50 nonsmoking adults who were recruited through open advertisements on the Internet and at local universities. A screening questionnaire was used to verify eligibility among subjects (nonsmoking adults, 18–50 years of age, without respiratory diseases such as ever asthma or having had a cold in the preceding 3 weeks). Participants were requested to avoid swimming pools during the week before the session and to avoid taking a shower the day of the swimming experiment. The study was approved by the ethics committee of the research center following the international regulations, and all volunteers signed an informed consent before participation.

**Figure 1:**
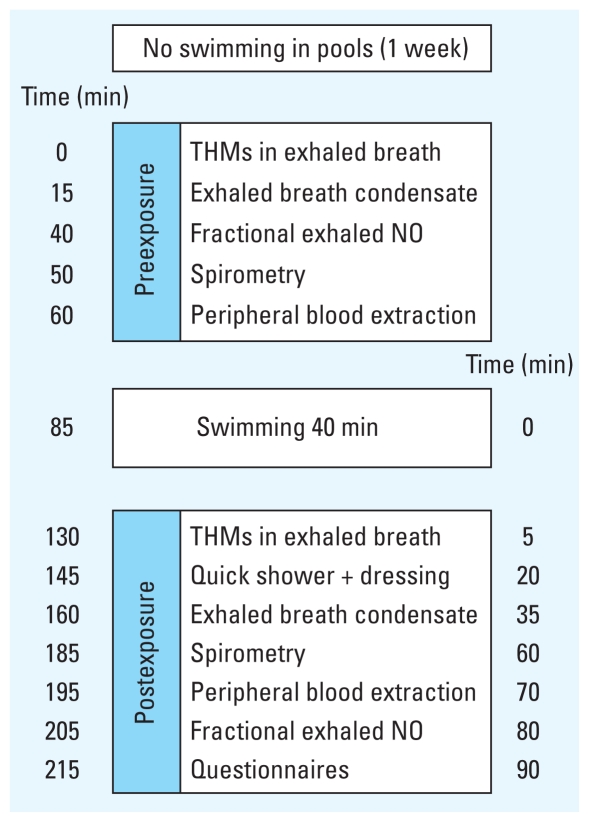
Study design and timing of the sample collection and *in situ* measurements. Urine was also collected for genotoxicity analysis (Kogevinas et al. 2010).

A single, indoor, 25-m-long chlorinated swimming pool in Barcelona, Spain, was used for the study. Every day, one to four participants were evaluated between 0900 and 1400 hours (before lunch) in May, June, September, or October 2007. Before and after the subjects swam in the chlorinated pool for 40 min, a battery of measurements and biological samples was collected to evaluate respiratory biomarkers according to a strict schedule (Figure 1). Biological samples and measurements before and after the swim were obtained in a room inside the sports center where the swimming pool was located but separated from the swimming pool area.

### Respiratory biomarkers

#### 8-Isoprostane and cytokines

EBC was obtained approximately 70 min before swimming began and 35 min after swimming ended using an EcoScreen condenser (Jaeger GmbH, Würzburg, Germany) following American Thoracic Society/European Respiratory Society Task Force recommendations (Horvath et al. 2005). Samples were obtained through breathing at normal frequency and tidal volume until a total expiratory volume of 180 L was achieved. After collection, the condensing device was centrifuged at 4°C, and the resultant total EBC volume (~ 4 mL) was transferred into Eppendorf tubes and rapidly frozen in liquid nitrogen. All samples were lyophilized and stored at −80°C before analysis. 8-Isoprostane was analyzed through an enzyme-linked immunosorbent assay (ELISA; Cayman Chemical, Ann Arbor, MI, USA). Using the BD Cytometric Bead Array (CBA; BD Biosciences, Erembodegem, Belgium) and the BD FACSCalibur Flow Cytometer (Becton Dickinson, San Jose, CA, USA), a particle-based immunoassay, we determined levels of the following eight cytokines and a growth factor: RANTES (regulated upon activation, normal T-cell expressed, and secreted), vascular endothelial growth factor (VEGF), tumor necrosis factor (TNF), interleukin (IL) 12p70, IL-4, IL-8, IL-10, interferon-gamma (IFN-γ), and IFN-γ–induced protein 10 (Ip10). Levels were characterized as picograms per milliliter of EBC.

#### CC16 and surfactant pneumoprotein D (SP-D)

Two 5-mL Vacutainer serum tubes were collected from each participant by venipuncture before swimming and 70 min after swimming. Samples were centrifuged at 2,500 rpm for 15 min, and serum was subsequently distributed in 1.8-mL aliquots and stored at −80°C. CC16 and SP-D were analyzed by ELISA using commercial kits (Biovendor Laboratorní medicína a.s., Modrice, Czech Republic). Intra- and interassay coefficients of variation ranged from 2.0% to 2.5% in both cases for serum SP-D and from 4.0% to 5.0% in both cases for serum CC16. The minimum detectable concentration in serum was set at 0.2 ng/mL for SP-D and 20 pg/mL for CC16 (Biovendor Laboratorní medicína a.s.). Levels were expressed as micrograms per liter of serum.

#### FeNO

FeNO was measured 40 min before and 80 min after swimming with an electrochemical portable device (NIOX-MINO; Aerocrine, Solna, Sweden), with a constant airflow rate of 50 mL/sec. Duplicate measurement was performed in 50% of the participants to evaluate reproducibility, resulting in a coefficient of variation of 9.7% (SD = 10.6). Levels were expressed as parts per billion.

#### Lung function

Forced expiratory volume in 1 sec (FEV_1_) and forced vital capacity (FVC) were measured 30 min before and 60 min after participants swam, with an EasyOne portable spirometer (ndd Medical Technologies, Zürich, Switzerland) following standard recommendations (Miller et al. 2005). FEV_1_ and FVC were expressed as the percentage from the predicted value by age, sex, and height (Roca et al. 1986).

### Biomarkers of exposure

The four THMs—chloroform, bromodichloromethane, dibromochloromethane, and bromoform—were measured in exhaled breath before swimmers entered the swimming pool (80 min before swimming) and just after they swam (5 min after leaving the pool) (Figure 1), as markers of individual exposure to DBPs. Exhaled breath samples were collected using a portable system for end-exhaled breath sampling, which has been described previously (Lourencetti et al. 2010). Briefly, volunteers were required to breathe through the sampling device equipped with an adsorption cartridge packed with Tenax TA (Supelco, Bellefonte, PA, USA). A total volume of 1 L was collected per person. The air passed through a stainless-steel cartridge (0.5 cm diameter and 9 cm long) containing 1.8 g Tenax TA (60/80 mesh). Chloroform, bromodichloromethane, dibromochloromethane, and bromoform were determined by an Automatic Thermal Desorption System (ATD 400; Perkin-Elmer, Shelton, CT, USA) coupled to an Autosystem gas chromatograph with electron capture detection (Perkin-Elmer). Concentrations were expressed as micrograms per cubic meter.

### Environmental measurements

Environmental measurements were taken to characterize the swimming pool and to complement the exposure assessment to DBP. Free chlorine, THMs, and mono-, di-, and trichloramine were measured in pool water. A 1-L composite water sample was collected at four different points of the pool for each participant while he or she was swimming. A single value for free chlorine, monochloramine, dichloramine, and trichloramine was obtained for each participant as measured by *N*,*N*-diethyl-*p*-phenylenediamine (DPD) procedure with a portable photometer (DINKO Instruments, Inc., Barcelona, Spain). The methods for water and air THM analyses have been described elsewhere (Lourencetti et al. 2010). Briefly, for THM analyses, 5 mg sodium thiosulfate was added to a 40-mL glass vial with screw cap and polytetrafluoroethylene-lined silicone septa. Water samples were stored at 4°C until laboratory analysis on the same day. THMs in water were determined using a SOLATek 72 Multi-Matrix Vial Autosampler (Tekmar Dohrmann, Mason, OH, USA) coupled to a purge-and-trap concentrator (Tekmar 3100; Tekmar, Cincinnati, OH, USA), which transfers the sample directly to a gas chromatograph coupled to a mass spectrometer (Voyager MS; ThermoQuest Finnigan, Manchester, UK) and had a coefficients of variation between 0.98% and 5.6%. An indoor air sample for THM measurements was collected for each participant with a pump located 60 cm above the floor and 1.5 m from the pool border, at 7 mL/min flow rate for 20 min through an adsorption cartridge filled with Tenax TA. Quality control was assured by daily calibration of the pump. The four THMs were measured as described for exhaled breath samples and were expressed as micrograms per cubic meter.

Additional air samples were collected to measure trichloramine in a subset of the days (6 days). Air was collected with a constant flow sampling pump (flow rate of 1.2 L/min for an mean ± SD of 115 ± 32 min), within 1 m from the water and at a height of 60 cm above the floor level. The instrumental analyses were performed at the Institute for Risk Assessment Sciences at Utrecht University (Utrecht, the Netherlands) following the method described by Hery et al. (1995); further details are available elsewhere (Jacobs et al. 2007). Trichloramine measurements were used for comparison with other swimming pools but were not used as personal exposure estimates because only 2 of the 6 trichloramine measurement days coincided with the experimental study involving only two participants.

### Other information collected

Questionnaires were used to collect information on personal and family history of atopic diseases, exposure to environmental tobacco smoke, diet, sociodemographic data, frequency and duration of swimming pool attendance and other physical activity, and way of commuting to the swimming pool facility. Weight and height were measured with standard procedures. Exercise intensity during swimming was estimated using the distance swum by each participant during the 40 min. Energy expenditure (in kilocalories) was estimated using the swimming speed and the weight of the participant, assuming that swimming at 46 m/min equals 11 metabolic equivalent tasks (METs; kilocalories per kilogram per hour):


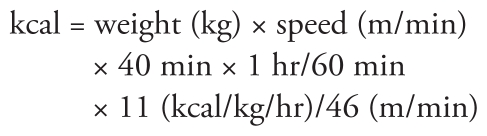


(Ainsworth et al. 2000). Atopic status was measured with the Phadiatop test (Pharmacia & Upjohn, Uppsala, Sweden), a qualitative test for serum-specific immunoglobulin E to a mixture of common allergens (Vidal et al. 2005). A single-nucleotide polymorphism in the *CC16* gene (rs3741240), known to modify gene expression, was genotyped using Sequenom (CEGEN-Santiago, San Diego, CA, USA). DNA was extracted from peripheral blood samples.

### Statistical analysis

The distribution of each biomarker was evaluated with a test for normality evaluating skewness and kurtosis. Mean or median values were reported accordingly to describe central tendencies. We calculated the individual change in the concentration of each biomarker after swimming in the pool (concentration after – before). Samples with cytokines under the detection limit before (30.1%) or after (27.1%) swimming were imputed half the detection limit. Those with undetectable levels before and after swimming were excluded from the statistical analysis. A nonparametric test was used to evaluate whether there was a significant change in the concentration of each biomarker. Linear regression models were fitted to calculate the association between changes in the concentration of each respiratory biomarker and the personal markers of DBP exposure and exercise intensity. The β-coefficient of a change in a unit in the concentration of each respiratory marker was calculated for an increase from the 25th percentile to the 75th percentile in the exposure parameters. All covariates were tested in each model, and only those that were statistically significant were retained in the multivariate models. The *p*-value threshold for statistical significance was set up at < 0.05. Interactions were tested by introducing the product of the variables in the regression model. All the statistical analyses were performed with the statistical package STATA 8.2 (StataCorp., College Station, TX, USA).

## Results

We recruited 50 subjects for the study. We excluded two subjects with history of asthma for the present analysis, resulting in a sample of 48 subjects. Most participants were women (65%) and were highly educated (92% with university studies), with an average age (± SD) of 30 ± 6.1 years, and 30% were positive to the Phadiatop test. Twenty percent were regular swimmers (at least once per month), and 54% practiced sport at least once a week. Regarding the *CC16* genotype (A38G), frequencies were 39%, 12%, and 49% for AG, AA, and GG, respectively. The genotyping frequency was 91%, and it was in Hardy–Weinberg equilibrium (*p* = 0.365). Minor allele frequencies were similar to those described in the International HapMap Project for European individuals (International HapMap Consortium 2003). The mean (± SD) speed during swimming was 22.5 ± 9.7 m/min, and the mean energy expenditure was 248.5 ± 120.6 kcal. We had one missing value for energy expenditure, one for THM in exhaled breath, one for FeNO, and three for 8-isoprostane in EBC.

Average free chlorine level in the pool water was 1.17 ± 0.4 mg/L. Average total THM concentration in water was 45.4 ± 7.3 μg/L (Table 1). The mean (± SD) level of THM in exhaled breath before swimming was 1.19 ± 0.40 μg/m^3^ for total THMs, 0.72 ± 0.28 μg/m^3^ for chloroform, 0.25 ± 0.09 μg/m^3^ for bromodichloromethane, 0.13 ± 0.06 μg/m^3^ for dibromochloromethane, and 0.10 ± 0.07 μg/m^3^ for bromoform. After swimming, THMs in exhaled breath increased on average about seven times. The increase was similar by age group, sex, or body mass index (data not shown). Chloroform levels in exhaled breath were significantly correlated with levels in the swimming pool’s air, but not with levels in water (Table 2). Dichloramine in water was inversely and significantly correlated with brominated THMs but not with chloroform in water, air, and exhaled breath. Free chlorine in water was not significantly correlated to total THMs in water but was significantly correlated to total THMs in air and exhaled breath. The energy expenditure correlated significantly only with bromoform concentration in exhaled breath after swimming. Trichloramine in water was undetectable, and monochloramine correlated with the same DBPs as dichloramine, so we show only dichloramine in the tables.

**Figure 2:**
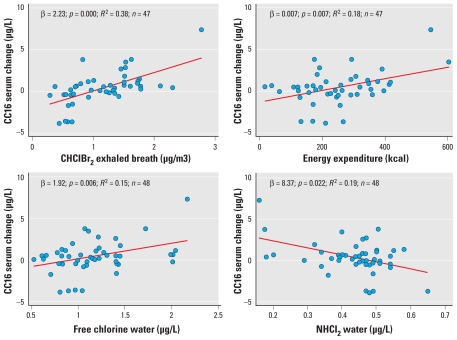
Correlation between the change in serum CC16 concentration and dibromochloromethane (CHClBr_2_) in exhaled breath, energy expenditure during swimming, free chlorine in water, and dichloramine (NHCl_2_) in water.

The concentration of CC16 in serum was increased significantly after swimming, with an overall median increase of 0.47 μg/L (3.3% increase) (Table 3). We detected no significant changes in percent predicted FEV_1_, percent predicted FVC, FEV_1_/FVC, FeNO, serum SP-D, 8-isoprostane (Table 3), or cytokines in EBC (Table 4). The increase in serum CC16 concentration was significantly correlated with different indicators of DBP exposure (negatively with dichloramine in water and positively with free chlorine in water and bromodichloromethane, dibromochloromethane, and bromoform in exhaled breath) and with energy expenditure (Table 3, Figure 2). In multivariate models, both energy expenditure and markers of DBP exposure remained significantly associated with the increase in CC16 after mutual adjustment (Table 5). An interquartile range (IQR) increase in energy expenditure was associated with a significant increase in 8-isoprostane in EBC after swimming. We found an interaction with the change in 8-isoprostane and swimming regularly (*p*-value = 0.04). 8-Isoprostane decreased among those who swam regularly (median change, −1.0 pg/mL; SD = 1.2; *p*-value = 0.04), whereas it tended to increase among those who did not swim regularly (0.62 pg/mL; SD = 2.1; *p*-value = 0.09).

**Figure 3:**
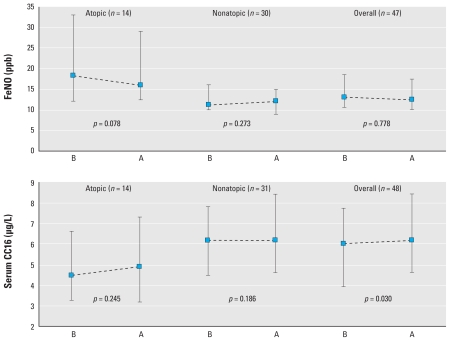
Concentration of FeNO and serum CC16 before (B) and after (A) swimming, stratified by atopic status (median and IQR). The Phadiatop test was used to define atopic status. *p*-Value from a Mann–Whitney test between atopics and nonatopics: 0.022 for FeNO and 0.560 for CC16.

When we calculated the change in the biomarker concentration as a relative measure [(levels after – before)/levels before], we found the same patterns. Bivariate analyses showed that the changes in the levels of these respiratory biomarkers did not differ by sex, age, body mass index, or the time spent in active commuting (walking or cycling) to the swimming pool facility. Atopic participants had higher baseline FeNO concentrations than did nonatopic participants, and they tended to have a decrease in FeNO after swimming, whereas nonatopic subjects remained stable (Figure 3). The increase in CC16 concentration in serum was not modified significantly by atopic status. Furthermore, CC16 change was not different among CC16 genotypes, modeled as dichotomous (GG vs. AA/AG; *p*-value = 0.507) (data not shown).

## Discussion

We detected a slight but significant increase in lung epithelial permeability, as estimated by serum CC16, in healthy adult volunteers after swimming in a chlorinated pool. Energy expenditure during swimming and change in THM concentrations in exhaled breath after swimming (indicating higher DBP exposures) were significant predictors of increases in serum CC16, suggesting that these exposures may have contributed to an increase in lung permeability. We observed no significant changes in lung function tests or markers of inflammation or oxidative stress in adults after swimming in a chlorinated pool.

The lack of an association between swimming and lung function and FeNO was consistent with previous studies with a comparable design (Carbonnelle et al. 2002, 2008; Moreira et al. 2008; Pedersen et al. 2009). However, evidence for serum CC16 is less consistent. Serum CC16 did not vary significantly in 11 young adults (Carbonnelle et al. 2008) and in 16 children (Carbonnelle et al. 2002), whereas it decreased (29% decrease) among 13 adults after swimming in a chlorinated pool, with a concentration of trichloramine in air between 160 and 280 μg/m^3^ in one study (Carbonnelle et al. 2008) and of 490 μg/m^3^ in the other (Carbonnelle et al. 2002). Carbonnelle et al. (2002) detected an increase in serum CC16 levels among 14 elite swimmers after they swam in a chlorinated pool (44% increase) and a nonchlorinated pool (52% increase), suggesting that the hyperpermeability of the lung epithelium after swimming could be caused by physical activity (Nanson et al. 2001). We showed in the present study that the both exercise intensity during swimming and markers of DBP exposure were associated with the increase in serum CC16 after mutual adjustment, supporting the hypothesis of independent effects of exercise and chemical exposure on the permeability of the lung epithelium.

The unchanged concentration of 8-isoprostane after swimming suggests the lack of association with oxidative stress in the airways. However, 8-isoprostane tended to increase with energy expenditure, in accordance with a previous study showing that oxidative stress increases after physical exercise in healthy subjects (Moller et al. 1996). We detected no changes in the eight cytokines and one growth factor measured in EBC, in accordance with a previous study that did not find changes in other markers of inflammation in 21 adolescents after they swam in a chlorinated pool (Pedersen et al. 2009). Although the concentrations of cytokines in EBC of our healthy study population were relatively low (~ 1 pg/mL), they were detectable in most samples (~ 80%). However, partly because of the lack of appropriate reference values, the validity of using these proteins in EBC as markers of acute inflammation in healthy subjects needs to be determined.

The present study has a larger sample size than previous studies with a similar design [*n* = 48, vs. 30 (Moreira et al. 2008), 29 (Carbonnelle et al. 2002), 21 (Pedersen et al. 2009), and 11 (Carbonnelle et al. 2008)]. However, statistical power could still be limited for detecting minor changes in some biomarkers with statistically significance. We selected the timing of sample collection to account for the specific expression dynamics of the different biomarkers and highly controlled this timing during field work. However, available data on expression dynamics of some biomarkers were limited or inconsistent. For example, an increase in FeNO has been observed right after swimming (Carbonnelle et al. 2002) and also 6 hr after mold exposure (Stark et al. 2005). Therefore, undetected changes in some biomarkers due to inappropriate sample timing cannot be ruled out. Although THMs are not irritants and are not likely the putative agents for the respiratory effects associated to the swimming pool environment, we used their occurrence in exhaled breath as a surrogate for DBP dose because THMs are the most prevalent DBPs in swimming pools and are easy to measure in exhaled breath. The observed dichloramine concentrations (0.43 mg/L; Table 1) in water were low compared with other swimming pools using chlorine for disinfection (Weaver et al. 2009). Furthermore, we used the same DPD method for all participants; therefore, the influence of the biases should be minimal. The season when we conducted the study (spring–summer) probably represented lower levels of DBP exposures than would the rest of the year because the facility was highly ventilated with doors and windows opened. Trichloramine in the air ranged from 0.17 to 0.43 mg/m^3^ (mean, 0.29 mg/m^3^), which is below the World Health Organization (2006) recommendations of 0.5 mg/m^3^ but comparable to the study by Carbonnelle et al. (2008).

The measurement of a battery of respiratory biomarkers allowed us to explore short-term respiratory changes that may reflect different mechanisms of respiratory effect in relation to swimming pool exposure. However, there is limited knowledge of the clinical significance to interpret the health impacts of the biomarkers measured. The present study replicates with a higher sample size the methods of previous studies and provides new evidence on biomarkers not measured previously, including markers in EBC. It suggests for the first time that atopic status and *CC16* genotype do not modify the effect of swimming in a pool on the permeability of the lung epithelium. Furthermore, we attempted to disentangle the effects of chemical exposure and exercise by measuring individual exposure to DBPs and energy expenditure during swimming.

The increase in serum CC16 after swimming in a well-maintained and highly ventilated indoor swimming pool confirms the high sensitivity of this assay to detect subtle changes in the concentration of this biomarker after environmental exposures. The fact that we detected no differences in serum CC16 levels by *CC16* genotype further supports the hypothesis that the association is attributable to an increased permeability of the lung epithelium rather than an increase in CC16 synthesis, which may differ by genotypes (Laing et al. 2000). The higher molecular weight of SP-D (130 kDa) (Kishore et al. 2006) compared with CC16 (16 kDa) (Broeckaert et al. 2000) probably explains the lack of increase in serum concentration of this protein after swimming because its higher molecular weight would not permit the passive diffusion of the molecule through the epithelium barrier. Previous studies that measured other surfactant proteins (SP-A and SP-B) in serum in similar settings found inconsistent results (Carbonnelle et al. 2002, 2008).

We assessed the role of atopy as an effect modifier because some epidemiologic studies have reported an increased asthma risk for swimming pool attendance among atopic children (Bernard et al. 2006, 2007, 2008, 2009). Baseline serum CC16 levels and the change after swimming were similar among atopics and nonatopics. Atopic status did not modify the effect of swimming pool exposure on the markers studied or on pulmonary function, in agreement with a previous study (Bonetto et al. 2006). However, atopic participants had higher baseline FeNO levels, and atopy modified the effect of swimming (*p* = 0.02). FeNO remained unchanged among nonatopics, whereas it tended to decrease among atopics. Moreira et al. (2008) found no changes on FeNO that did not vary by atopic status or asthma in 30 competitive swimmers after a training session.

Among the battery of respiratory biomarkers evaluated, only serum CC16 levels changed significantly after swimming. Given the moderate increase detected (3.3%), the high variability in CC16 levels in healthy subjects (Broeckaert et al. 2000), and the lack of reference values of CC16, the clinical relevance of this short-term effect is unclear (Carbonnelle et al. 2008), and further studies are necessary to establish the health impacts of short-term serum CC16 changes (Broeckaert et al. 2000; Lakind et al. 2007). Further, previous studies have shown that this acute increase in serum CC16 is transient and that serum CC16 returns to baseline levels a few hours after exposure ceases (Carbonnelle et al. 2002, 2008). We interpret the increase in lung epithelial permeability after swimming as an acute physiological reaction of the lung caused by exercise and exposure to some DBPs. Long-term effects cannot be extrapolated from these results until the clinical and physiological relevance of CC16 short-term change is understood further.

Our exposure-assessment data have been used in a companion paper in this issue by Kogevinas et al. (2010), who showed that swimming produced genotoxic effects that were associated with the concentrations of brominated THMs, but not chloroform, in the swimmers’ exhaled breath. This finding was supported by another companion paper in this issue by Richardson et al. (2010), who identified > 100 DBPs in the pool water and showed that the water was mutagenic.

In summary, we detected a slight increase in serum CC16, which is a marker of lung epithelium permeability, in healthy adults after 40 min of swimming in an indoor chlorinated pool. Exercise and DBP exposure explained this association, without involving further inflammatory mechanisms. Further research is needed to confirm the results, disentangle the effects of exercise and DBP exposure, establish the clinical relevance of short-term serum CC16 changes, and evaluate the long-term respiratory health impacts of swimming.

## Figures and Tables

**Table 1:** Physicochemical parameters in water, air, and exhaled breath and exercise intensity performed by participants (*n* = 48).

Measurement	Mean ± SD	Median	Minimum	Maximum
Water				
Free chlorine (mg/L)	1.17 ± 0.4	1.10	0.5	2.17
Dichloramine (mg/L)	0.43 ± 0.1	0.46	0.16	0.65
Temperature (°C)	27.2 ± 0.4	27.4	26.5	27.7
pH	7.3 ± 0.1	7.3	6.9	7.5
Chloroform (μg/L)	16.1 ± 3.4	16.7	8.5	20.8
Bromodichloromethane (μg/L)	12.3 ± 2.3	11.9	9.3	22.8
Dibromochloromethane (μg/L)	10.9 ± 3.1	10.5	6.5	22.6
Bromoform (μg/L)	6.1 ± 2.4	5.7	3.0	16.2
Total THMs (μg/L)	45.4 ± 7.3	45.5	35.2	75.2

Air				
Chloroform (μg/m^3^)	35.0 ± 12.3	31.4	19.5	61.6
Bromodichloromethane (μg/m^3^)	14.6 ± 5.0	13.0	7.5	23.4
Dibromochloromethane (μg/m^3^)	13.2 ± 4.3	12.4	6.0	26.2
Bromoform (μg/m^3^)	11.2 ± 5.2	8.4	4.4	22.6
Total THMs (μg/m^3^)	74.1 ± 23.7	68.9	44.0	124.9

Exhaled breath (after swimming)^a^				
Chloroform (μg/m^3^)	4.5 ± 1.7	4.6	1.1	8.1
Bromodichloromethane (μg/m^3^)	1.8 ± 0.5	1.6	0.7	3.2
Dibromochloromethane (μg/m^3^)	1.2 ± 0.5	1.2	0.3	2.8
Bromoform (μg/m^3^)	0.5 ± 0.2	0.4	0.1	1.3
Total THMs (μg/m^3^)	7.9 ± 2.8	7.7	2.3	14.0

Exercise intensity^a^				
Distance swam (km)	0.90 ± 0.4	0.95	0.05	1.75
Energy expenditure (kcal)	248.5 ± 120.6	241.9	16.8	603.3

a *n* = 47.

**Table 2:** Spearman correlation coefficients (*r*) between the different exposure indicators measured (*n* = 47).

Medium (concentration)	Water (μg/L)		Concentration in exhaled breath after swimming (μg/m^3^)					Energy expenditure (kcal)^a^
	Free Cl	NHCl_2_	CHCl_3_	CHCl_2_Br	CHClBr_2_	CHBr_3_	TTHMs	
Water (μg/L)								
Free Cl			0.24	0.34*	0.48*	0.27	0.35*	0.16
NHCl_2_	−0.28		−0.22	−0.44*	−0.44*	−0.50*	−0.37*	−0.26
CHCl_3_	−0.24	0.17	0.27	−0.03	−0.32	0.01	0.07	−0.04
CHBr_3_	−0.03	−0.31*	−0.40*	−0.05	0.25	0.24	−0.17	0.08
TTHMs	−0.04	−0.40*	0.06	0.19	0.18	0.48*	0.14	0.04

Air (μg/m^3^)								
CHCl_3_	0.19	0.04	0.64*	0.40*	0.13	0.29*	0.52*	−0.001
CHBr_3_	0.24	−0.29*	0.22	0.35*	0.29	0.55*	0.30*	0.12
TTHMs	0.38*	−0.13	0.55*	0.48*	0.34*	0.43*	0.53*	0.03

Exhaled breath after (μg/m^3^)								
CHCl_3_				0.83*	0.60*	0.55*	0.94*	0.14
CHCl_2_Br					0.80*	0.72*	0.94*	0.18
CHClBr_2_						0.74*	0.79*	0.18
CHBr_3_							0.70*	0.32*
TTHMs								0.19

Abbreviations: CHBr_3_, bromoform; CHClBr_2_, dibromochloromethane; CHCl_2_Br, bromodichloromethane; CHCl_3_, chloroform; NHCl_2_, dichloramine; TTHMs, total THMs.

a Kilocalories expended during the 40 min.

* *p* < 0.05.

**Table 3:** Level of respiratory markers before and after swimming: linear regression coefficients of the change after swimming for the exposure parameters.

Parameter	Percent predicted		FEV_1_/FVC	FeNO (ppb)	8-Isoprostane (pg/mL)	SP-D (μg/L)	CC16 (μg/L)
	FEV_1_	FVC					
*n*	48	48	48	47	45	48	48
Median (IQR)							
Before	97.3 (90.1 to 103.6)	98.1 (90.0 to 105.4)	0.83 (0.8 to 0.9)	13 (10.5 to 18.5)	1.6 (1.0 to 2.0)	54.4 (39.3 to 68.0)	6.01 (3.9 to 7.7)
After	96.3 (90.4 to 105.1)	95.9 (90.7 to 104.2)	0.83 (0.8 to 0.9)	12.5 (10 to 17.5)	1.3 (0.6 to 2.5)	55.1 (45.4 to 85.0)	6.21 (4.6 to 8.4)
Change	−0.6 (−2.5 to 2.4)	−2.0 (−5.1 to 3.9)	0.0 (−0.02 to 0.04)	0.0 (−2 to 2)	−0.03 (−0.8 to 1.1)	1.0 (−3.7 to 6.6)	0.47 (−0.3 to 1.1)
* p*-Value (change ≠ 0)^a^	0.83	0.46	0.24	1.00	0.91	0.44	0.03
Change in respiratory markers for an increase from 25th to 75th percentile in exposure parameters (95% confidence interval)^b^							
Free chlorine to water (mg/L)	0.54 (−0.90 to 1.99)	−2.44 (−5.77 to 0.88)	0.02 (0.00−0.04)*	−1.22 (−2.51 to 0.06)	0.61 (−0.17 to 1.39)	0.58 (−6.26 to 7.42)	1.02 (0.30−1.74)**
NHCl_2_ to water (μg/L)	−0.50 (−1.59 to 0.57)	0.12 (−2.42 to 2.67)	−0.01 (−0.02 to 0.01)	0.92 (−0.03 to 1.91)	−0.10 (−0.71 to 0.51)	2.93 (−2.10 to 7.97)	−0.84 (−1.38 to −0.33)**
CHCl_3_ to exhaled breath (μg/m^3^)	0.48 (−0.78 to 1.74)	0.73 (−2.25 to 3.72)	0.00 (−0.02 to 0.02)	0.14 (−1.07 to 1.35)	0.59 (−0.17 to 1.32)	0.34 (−5.69 to 6.37)	0.24 (−0.44 to 0.92)
CHCl_2_Br to exhaled breath (μg/m^3^)	0.14 (−0.77 to 1.06)	−0.43 (−2.60 to 1.73)	0.00 (−0.01 to 0.02)	0.22 (−0.66 to 1.10)	0.43 (−0.13 to 0.99)	0.87 (−3.50 to 5.25)	0.55 (0.08 to 1.02)*
CHClBr_2_ to exhaled breath (μg/m^3^)	−0.05 (−1.79 to 1.69)	−1.07 (−5.18 to 3.03)	0.01 (−0.02 to 0.03)	0.22 (−1.43 to 1.88)	0.71 (−0.29 to 1.70)	1.06 (−7.22 to 9.35)	1.92 (1.19 to 2.67)**
CHBr_3_ to exhaled breath (μg/m^3^)	0.35 (−0.97 to 1.65)	−0.05 (−3.16 to 3.06)	0.00 (−0.02 to 0.02)	−0.07 (−1.53 to 1.38)	0.32 (−0.41 to 1.05)	3.13 (−3.09 to 9.30)	1.21 (0.59 to 1.82)**
TTHMs to exhaled breath (μg/m^3^)	0.31 (−0.79 to 1.44)	0.17 (−2.49.2.81)	0.00 (−0.02 to 0.02)	0.17 (−0.93 to 1.24)	0.54 (−0.12 to 1.20)	0.76 (−4.57 to 6.10)	0.59 (0.02 to 1.18)*
Energy expenditure (kcal)	−0.65 (−2.03 to 0.73)	−2.97 (−6.10 to 0.17)	0.02 (−0.00 to 0.04)	0.20 (−1.14 to 1.54)	0.98 (0.22 to 1.74)*	0.85 (−5.73 to 7.41)	1.04 (0.37 to 1.73)**

Abbreviations: CHBr_3_, bromoform; CHClBr_2_, dibromochloromethane; CHCl_2_Br, bromodichloromethane; CHCl_3_, chloroform; NHCl_2_, dichloramine; TTHMs, total THMs. Percent predicted refers to percentage of that predicted by age to sex to and height. SP-D and CC16 were measured in serum. 8-Isoprostane was measured in EBC.

a Wilcoxon test.

b β-Coefficients from linear regression models represent a change in the biomarker level for an increase from 25th to 75th percentile of the exposure parameter. FeNO models are adjusted for rhinitis; 8-isoprostane models are adjusted for usual swimming pool attendance. The other models are crude.

* *p* < 0.05.

** *p* < 0.01.

**Table 4:** Concentration of eight cytokines and VEGF (pg/mL) in EBC before and after swimming.

Median (IQR)	RANTES	Ip10	VEGF	TNF	IL-12p70	IL-10	IL-8	IFN-γ	IL-4
*n*^a^	31	36	44	39	39	43	40	32	38
Before	0.85 (0.0 to 2.0)	1.18 (0.1 to 2.5)	3.63 (1.5 to 8.2)	0.89 (0.4 to 1.7)	0.66 (0.0 to 1.0)	0.89 (0.2 to 1.5)	1.24 (0.7 to 2.2)	1.29 (0.0 to 1.8)	0.70 (0.3 to 2.0)
After	1.17 (0.4, 1.6)	1.33 (0.0 to 2.2)	4.41 (2.3 to 7.1)	0.71 (0.4 to 1.6)	0.31 (0.0 to 0.7)	0.84 (0.0 to 1.5)	1.42 (0.4 to 2.2)	1.25 (0.0 to 2.4)	0.72 (0.3 to 1.1)
Change	−0.20 (−0.8, 1.1)	−0.08 (−1.3 to 1.3)	0.11 (−3.4 to 4.0)	−0.16 (−0.7 to 0.7)	0.00 (−0.6 to 0.0)	−0.15 (−0.6 to 0.7)	0.00 (−0.7 to 0.4)	−0.02 (−1.5 to 1.5)	−0.07 (−1.2 to 0.7)
*p*-Value (change ≠ 0)^b^	0.631	0.683	0.879	0.477	0.107	0.740	0.898	0.903	0.658

a Samples with undetectable levels were imputed half of the detection limit; participants with undetectable levels before and after swimming were excluded.

b Wilcoxon test.

**Table 5:** Multiple linear regressions between serum CC16 concentration (μg/L) in relation to a unit increase in indicators of DBP exposure and energy expenditure.

Model	Variables	β (95% CI)	*p*-Value	*R*^2^	*n*
1	CHClBr_2_, exhaled breath (μg/m^3^)	1.68 (0.93 to 2.43)	< 0.001	0.45	46
	Energy expenditure (kcal)	0.69 (0.09 to 1.28)	0.024		

2	CHClBr_2_, exhaled breath (μg/m^3^)	1.49 (0.65 to 2.33)	0.001	0.46	46
	Energy expenditure (kcal)	0.68 (0.08 to 1.27)	0.027		
	Free chlorine (mg/L)	0.33 (−0.35 to 1.01)	0.336		

3	CHCl_2_Br, exhaled breath (μg/m^3^)	0.45 (0.01 to 0.89)	0.047	0.26	46
	Energy expenditure (kcal)	0.97 (0.30 to 1.64)	0.005		

4	CHCl_2_Br, exhaled breath (μg/m^3^)	0.35 (−0.09 to 0.78)	0.117	0.34	46
	Energy expenditure (kcal)	0.87 (0.22 to 1.51)	0.010		
	Free chlorine (mg/L)	0.77 (0.08 to 1.46)	0.030		

5	CHBr_3_, exhaled breath (μg/m^3^)	0.98 (0.31 to 1.65)	0.005	0.33	46
	Energy expenditure (kcal)	0.66 (−0.03 to 1.35)	0.060		

6	CHBr_3_, exhaled breath (μg/m^3^)	0.82 (0.16 to 1.48)	0.016	0.39	46
	Energy expenditure (kcal)	0.61 (−0.05 to 1.28)	0.070		
	Free chlorine (mg/L)	0.69 (0.03 to 1.36)	0.042		

7	TTHMs, exhaled breath (μg/m^3^)	0.46 (−0.10 to 1.02)	0.105	0.24	46
	Energy expenditure (kcal)	0.97 (0.29 to 1.65)	0.006		

8	TTHMs, exhaled breath (μg/m^3^)	0.30 (−0.25 to 0.86)	0.274	0.32	46
	Energy expenditure (kcal)	0.88 (0.22 to 1.53)	0.010		
	Free chlorine (mg/L)	0.79 (0.08 to 1.49)	0.030		

9	Free chlorine (mg/L)	0.85 (0.16 to 1.54)	0.017	0.28	47
	Energy expenditure (kcal)	0.90 (0.25 to 1.56)	0.008		

Abbreviations: CHClBr_2_, dibromochloromethane; CHCl_2_Br, bromodichloromethane; CHBr_3_, bromoform; CI, confidence interval; TTHMs, total THMs. No other variables were included in the models.
